# A new cultivar ‘Hisui no Kaori’ opens up a fragrant type of lettuce (*Lactuca sativa* L.)

**DOI:** 10.1270/jsbbs.24016

**Published:** 2024-08-27

**Authors:** Kousuke Seki, Masahiro Hiraga, Eri Soga, Kenji Matsui

**Affiliations:** 1 Nagano Vegetable and Ornamental Crops Experiment Station, 1066-1 Tokoo, Souga, Shiojiri, Nagano 399-6461, Japan; 2 Nagano Fruit Tree Experiment Station, 492 Ogawara, Suzaka, Nagano 382-0072, Japan; 3 Agriculture and Agricultural District Supporting Center in Suwa, Nagano, 1-1644-10 Kamigawa, Suwa, Nagano 392-0021, Japan; 4 Graduate School of Sciences and Technology for Innovation, Yamaguchi University, 1677-1 Yoshida, Yamaguchi 753-8515, Japan

**Keywords:** 2-acetyl-1-pyrroline, sweet fragrance, fragrant type, air temperature, delayed discoloration, stem lettuce, cooking

## Abstract

‘Hisui no Kaori’ is the first lettuce (*Lactuca sativa* L.) cultivar characterized by a sweet fragrance, attributed to 2-acetyl-1-pyrroline with the same compound as in fragrant rice and soybean cultivars, as well as edible leaves and stem. Field cultivation trials established optimal planting distances at 30 cm between seedlings, with a fertilizer requirement of N = 150 kg/ha. ‘Hisui no Kaori’ exhibited minimal stem burst as well as resistance to soft rot disease, proving easier to cultivate compared with prominent stem-type cultivars. Field cultivation tests at different altitudes and incubator tests revealed that an air temperature exceeding 20°C is pivotal for the development of the sweet fragrance. ‘Hisui no Kaori’ displayed moderately resistance to Fusarium wilt race 1 and highly resistance to race 2. In lettuce, discoloration is known to occur at the cut surface due to mechanical wounding. In a cut leaf test, ‘Hisui no Kaori’ was classified as having delayed discoloration. Overall, ‘Hisui no Kaori’ is expected to contribute to the expanding potential and the increasing market price of lettuce. This work represents a pioneering effort to open up the fragrant type of lettuce.

## Introduction

**Fig. 1.**
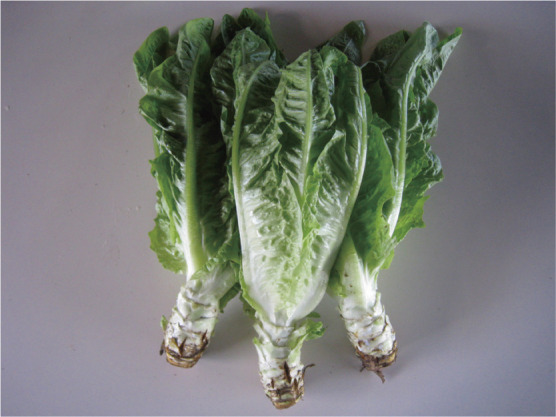
Image showing harvested ‘Hisui no Kaori’.

Lettuce (*Lactuca sativa* L.) is one of the most popular vegetables, with cultivars clearly distinguished by the edible parts, including leaf and stem types. The types with leaves as the edible part are the crisphead, leaf, butterhead, romaine, and Latin, and the stem is the edible part only in the stem type (Ryder 1999). Typically, lettuce is appreciated for its texture but lacks taste and fragrance. Interestingly, in our genetic resources, we identified a sweet fragrant lettuce. Hence, we aimed to break common sense by crossbreeding the sweetly fragrant stem-type ‘Kukichisya’ with Latin type ‘Rennet’ renowned for its leaf texture, to establish a new sweetly fragrant cultivar with edible leaves and stems. The result is the creation of the fragrant lettuce cultivar ‘Hisui no Kaori’ (Fig. 1) with edible leaves and stems as well as reduced stem burst. The meaning of the cultivar’s name is derived from the characteristics of the sweet fragrance and the bright green like jade. This study details the new cultivar’s agricultural productivity and phenotypic characteristics.

## Materials and Methods

### Breeding process

‘Hisui no Kaori’ was bred from a cross between ‘Kukichisya (stem type)’ with sweet fragrance and ‘Rennet (Latin type)’. From 2012 to 2019, the F_2_ to F_7_ generations were cultivated at the Nagano Vegetable and Ornamental Crops Experiment Station (Shiojiri City, Nagano Prefecture, Japan; 36° 10ʹ N, 137° 93ʹ E). Fragrance evaluation of fresh leaves, based on an organoleptic test, guided the selection of plants. In addition, wider leaf shape and thicker main stem played an important for the selection.

### Fragrance compound evaluation

‘Kukichisya’ and ‘Celtuce’ leaves were evaluated for fragrance using gas chromatography–mass spectrometry (GC-MS). Aerial plant organs (2.5 g fresh weight) were cut into pieces, and sealed in 22 mL glass vials (Perkin Elmer, Waltham, MA, USA), and frozen at –80°C for at least 24 h. The glass vials containing the plant material were immersed in a 25°C water bath for 10 min, and solid phase microextraction fiber (50/30 μm DVB/Carboxen/PDMS, Supelco, Bellefonte, PA, USA) was exposed to the headspace of the vial for 30 min at 25°C. Subsequently, the fiber-bound volatiles were analyzed using GC-MS system (QP-5050, Shimadzu, Kyoto, Japan) equipped with a 0.25 μm × 30 m Stabiliwax column (Restek, Bellefonte, PA, USA). To identify 2-acetyl-1-pyrroline (2AP), *Pandanus amaryllifolius* Roxb. leaves were analyzed under the same GC-MS conditions (Wakte *et al.* 2010). Confirmation of the compound’s identity involved verifying the matching retention time and MS profiles with the peak observed in ‘Kukichisya’.

### Field cultivation test

Stem lettuce cultivars, including ‘Hisui no Kaori’, ‘Celtuce’, and ‘Cologne’ were grown in the same fields at Nagano Vegetable and Ornamental Crops Experiment Station. Hand transplantation of seedlings into the mulch-covered field was conducted 15–20 days after sowing to trays. At the harvest stage, around 35–45 days after transplantation, total weight, adjusted weight, maximum leaf width, maximum leaf length, stem weight, maximum stem thickness, stem length, and occurrences of soft rot disease and stem burst were investigated.

To assess the influence of cultivation methods on ‘Hisui no Kaori’ growth and yield, seedlings were planted at intervals of 20, 25, and 30 cm, with three different fertilizer levels set at N = 100 kg/ha, 150 kg/ha, and 200 kg/ha (Tsuchiya 2009). The fertilizer used had a nitrogen (N):phosphate (P):potassium (K) ratio of 15:15:12. Cultivation tests were performed at production areas in Shiojiri and Kawakami.

### Organoleptic investigation in an incubator

‘Hisui no Kaori’ seedlings were examined one week after tray sowing to investigate the influence of air temperature on fragrance. Air temperature was set at four levels: 18°C, 21°C, 24°C, and 27°C. After 10 days, an organoleptic test was used to determine the presence of fragrance in seedlings.

### Infection assay for Fusarium wilt resistance

For *Fusarium oxysporum* f. sp. *lactucae* race 1 and 2 infection assays, the Japanese isolates SB1-1 and F-9501 were used, respectively. The detailed infection assay method was previously outlined in previous reports (Seki *et al.* 2020, 2021).

### Discoloration test using cut leaves

Postharvest leaves, cut into approximately 10 cm^2^ sections, were sealed in plastic bags and refrigerated at 4°C. Discoloration due to mechanical wounding was evaluated at 1, 3, 6, and 12 days after refrigeration. The discoloration index of leaf sections was as follows: 0, no browning and pinking; 1, partial browning or pinking; 2, substantial browning and pinking; 3, severe browning and pinking. Discoloration severity in leaf sections was calculated as follows:

Discoloration severity = (3A + 2B + C) × 100/(3N),

where A, B, and C represent the numbers of categories “3”, “2”, and “1” in the discoloration index, respectively, and “N” is the total number of leaf sections examined.

## Results

### Confirmation of fragrance compounds in ‘Kukichisya’

**Fig. 2.**
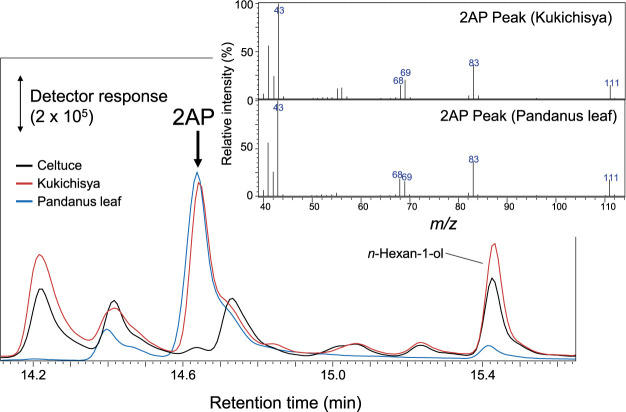
GC-MS chromatograms of fragrance compounds in *Pandanus* leaves, ‘Kukichisya’ and ‘Celtuce’, along with the MS profile of 2AP.

Validation of volatile compounds in ‘Kukichisya’ (fragrance) and ‘Celtuce’ (no fragrance) was performed through GC-MS. The volatile compounds of ‘Kukichisya’ exhibited significantly higher levels of detected compounds, including 3-methyl-butanal, 2AP, (Z)-(2)-hexen-1-ol, (–)-*β*-elemene, and octadecanal. Moreover, 3-methyl-butanal and 2AP were exclusively present in ‘Kukichisya’ and absent in ‘Celtuce’. Additionally, comparing the volatile compound data with that for *Pandanus* leaves confirmed the inclusion of 2AP in the fragrance profile of ‘Kukichisya’, establishing it as a key volatile compound imparting the cultivar’s characteristic fragrance (Fig. 2).

### Verification of the ‘Hisui no Kaori’ cultivation method

An investigation into the influence of planting density on the growth and yield of ‘Hisui no Kaori’ revealed that increasing spacing between seedlings led to heavier total and adjusted weights. Conversely, decreased spacing was associated with an increased rate of stem burst. Therefore, the results indicated that seedling spacing of 30 cm apart was optimal (Table 1). Regarding fertilizer amount, N = 20 kg/10 a inhibited stem elongation, suggesting that N = 15 kg/10 a was sufficient for cultivating ‘Hisui no Kaori’. Although ‘Celtuce’ and ‘Cologne’ showed soft rot disease in all instances, ‘Hisui no Kaori’ consistently exhibited soft rot disease-free harvests in all cases (Table 2).

### Cultivation tests in lettuce production areas

Cultivation of ‘Hisui no Kaori’ in Shiojiri and Kawakami from late May to early July revealed a sweet fragrance in plants cultivated at Shiojiri but not at Kawakami. To investigate this difference, we focused on the average air temperature during cultivation in these areas. Kawakami, at a higher altitude (1158 m), had an average air temperature of 15.2°C, whereas Shiojiri, at a lower altitude (740 m), showed an average air temperature of 20.3°C. An incubator test was also used to investigate the influence of air temperature on fragrance, with seedlings showing sweet fragrance when incubated at 27°C, 24°C, and 21°C but not 18°C. Therefore, the field and incubator test results consistently demonstrated fragrance at temperatures exceeding 20°C.

### Fusarium wilt resistance test

Cultivars were classified in terms of Fusarium wilt resistance based on the infected plants’ observed phenotypes. ‘Hisui no Kaori’ exhibited moderate resistance to race 1 and high resistance to race 2. In contrast, ‘Celtuce’ and ‘Cologne’ were susceptible to race 1 and highly resistant to race 2 (Table 3).

### Discoloration of cut leaves during storage

‘Romaine’ was used as a control cultivar for normal discoloration of cut leaves. Comparatively, ‘Hisui no Kaori’ exhibited delayed discoloration based on the discoloration severity index (Table 4).

## Discussion

In this study, we demonstrate that ‘Hisui no Kaori’ exhibits resistance to stem burst and soft rot disease, showing resistance against Fusarium wilt races 1 and 2 (Tables 1–3). Given that stem burst is a prevalent precursor to soft rot disease, resistance to stem burst is an important trait for stem-type lettuce. The use of resistant cultivars is the only effective control of the soil-borne Fusarium wilt. Resistance to races 1 and 2 is expected to result in stable production. Based on our cultivation tests, the optimal seedling spacing was 30 cm; at distances less than 30 cm, plant stems softened, triggering stem burst (Table 1). Furthermore, both field and incubator tests indicated that volatilization of the desired fragrance required an average air temperature exceeding 20°C during the cultivation period. This implies a limitation on the cultivation period, particularly from early to late summer, due to average air temperatures in fields located >1000 m above sea level at a major lettuce production area in Nagano Prefecture. In Japan, lettuce is grown outdoors throughout the year in varying production areas, but ‘Hisui no Kaori’ would not be suitable for a production area with low temperatures from autumn to spring. However, plant factories, with a constant high temperature throughout the year, and greenhouses, maintaining long-term high temperatures, are considered suitable for cultivating fragrant lettuce cultivars.

Fortuitously, ‘Hisui no Kaori’ was classified as a delayed discoloration cultivar, although selection for discoloration was not performed during the breeding process (Table 4). Mechanical wounding of leaves postharvest is known to induce discoloration on cut surfaces. Discoloration, considered a heritable trait influenced by a few quantitative trait loci (Simko *et al.* 2018), is an important postharvest trait. The green color of the inner stem is a distinctive and attractive feature of stem-type lettuce, particularly in the peeled and dried form known as ‘Yamakurage’ in Japan. Typically, ‘Yamakurage’ remains green, showing no browning or pinking, even in dried stems. This trait plays an important role in processed foods. The selection history of stem lettuce for maintaining green stems after peeling and drying suggests that ‘Hisui no Kaori’ has inherited the trait of reduced discoloration from stem lettuce. Moreover, the delayed discoloration trait could contribute to extending shelf life and reducing food loss.

**Fig. 3.**
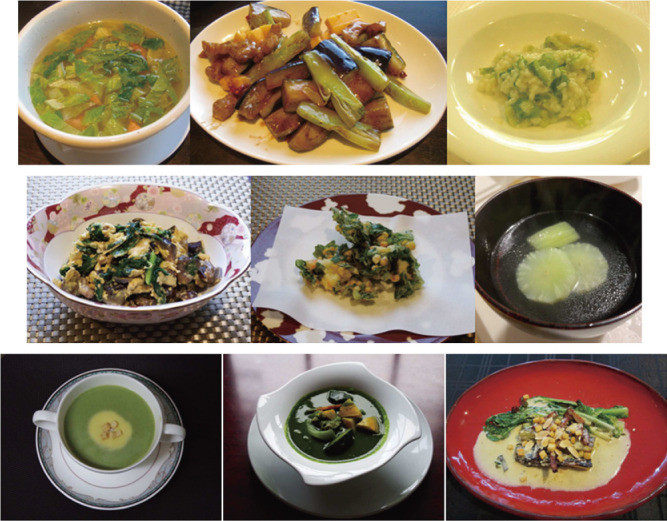
Various hot dishes based on ‘Hisui no Kaori’.

We have developed the fragrant type as a new category of lettuce by breeding the fragrant ‘Hisui no Kaori’, which has both edible leaves and stems and exhibits limited discoloration when cut. The detailed mechanisms and parental inheritance of the remarkable traits for disease resistance and a sweet fragrance are future concerns for lettuce breeding. Fragrance is considered a valuable trait in crops, with fragrant cultivars, such as fragrant rice and soybean cultivars, commanding high market prices (Kovach *et al.* 2009, Somta *et al.* 2019). Given that fragrance increases during heat-based cooking processes, such as baking and boiling (Somta *et al.* 2019), fragrant cultivars are likely to be treated as cooked vegetables. Therefore, ‘Hisui no Kaori’ may be used as a cooked vegetable rather than a salad vegetable. The jade green, which remains bright after cooking, is also one of the most attractive features for users. Indeed, this versatile vegetable finds utility in many dishes, including Japanese, Western, and Chinese cuisines (Fig. 3), and could contribute to expanding the potential of lettuce as a pioneer fragrant cultivar. ‘Hisui no Kaori’ has opened up an original way to create a new history of lettuce.

## Author Contribution Statement

KS designed the experiments. KS, MH, and ES conducted the field cultivation trials. KS performed Fusarium wilt resistance testing. KM performed GC-MS analysis of fragrance. KS, ES, and KM drafted the manuscript. All authors read and approved the final version of the manuscript.

## Figures and Tables

**Table 1.** Cultivation test results regarding the optimal ‘Hisui no Kaori’ planting distance. Twenty plants were investigated, using a row width of 45 cm. Seeding, planting, and parameter measurements were performed on July 10, August 1, and September 10, respectively

Distance between neighboring plants	Total weight	Adjusted weight	Maximum leaf width	Maximum leaf length	Stem weight	Maximum stem thickness	Stem length	Stem burst rate
(cm)	(g)	(g)	(cm)	(cm)	(g)	(cm)	(cm)	(%)
20	419	322	8.6	39.4	175	4.3	25.8	50
25	537	420	9.1	39.6	226	4.7	26.0	35
30	558	429	9.5	38.4	212	4.8	21.4	0

**Table 2.** Cultivation test results regarding the optimal fertilizer amount. Ten plants were investigated, using two replicates and a row width of 45 cm. Seeding, planting, and parameter measurements were performed on July 24, July 11, and August 21, respectively

Cultivar name	Fertilizer amount	Total weight	Adjusted weight	Maximum leaf width	Maximum leaf length	Stem weight	Maximum stem thickness	Stem length	Soft rot disease rate
	(kg/ha)	(g)	(g)	(cm)	(cm)	(g)	(cm)	(cm)	(%)
‘Hisui no Kaori’	N = 100	723	558	8.8	32.4	327	5.2	35.0	0
	N = 150	781	622	8.7	32.4	368	5.1	35.7	0
	N = 200	781	650	9.7	30.8	342	5.1	33.2	0
‘Celtuce’	N = 100	912	642	11.9	29.8	432	4.9	54.6	23.3
	N = 150	939	653	12.4	29.2	414	4.9	56.1	40.0
	N = 200	863	632	12.1	28.5	378	4.5	61.4	20.0
‘Cologne’	N = 100	852	580	6.7	36.2	364	4.3	65.9	3.3
	N = 150	866	603	6.9	35.3	376	4.4	60.2	13.3
	N = 200	829	599	7	34.3	368	4.2	61.9	6.7

**Table 3.** Infection assay to evaluate disease resistance against race 1 and 2 pathogens

Cultivar	Race 1				Race 2		
	No. of plants tested	Disease severity	Phenotype		No. of plants tested	Disease severity	Phenotype
‘Hisui no Kaori’	9	58.3	Moderate resistant		10	0	Highly resistant
‘Celtuce’	10	100	Susceptible		10	0	Highly resistant
‘Cologne’	10	100	Susceptible		10	0	Highly resistant
‘Patriot’	10	100	Susceptible		10	100	Susceptible
‘V lettuce’	9	2.8	Highly resistant		10	100	Susceptible
‘Banchu Red Fire’	10	100	Susceptible		10	0	Highly resistant

**Table 4.** Discoloration severity of cut leaves during storage

Cultivar	Days after harvest			
	1	3	6	12
‘Hisui no Kaori’	0	8.8	12.3	24.6
‘Romaine’	0	69	88.1	90.5
